# Rationale and design of FORTH: a randomised controlled trial assessing the effectiveness of HIV self-testing in increasing HIV testing frequency among gay and bisexual men

**DOI:** 10.1186/s12879-015-1300-6

**Published:** 2015-12-10

**Authors:** Muhammad S. Jamil, Garrett Prestage, Christopher K. Fairley, Kirsty S. Smith, John M. Kaldor, Andrew E. Grulich, Anna M. McNulty, Marcus Chen, Martin Holt, Damian P. Conway, Handan Wand, Phillip Keen, Colin Batrouney, Jack Bradley, Benjamin R. Bavinton, Dermot Ryan, Darren Russell, Rebecca J. Guy

**Affiliations:** The Kirby Institute, UNSW Australia, Sydney, NSW Australia; Australian Research Centre in Sex, Health and Society, La Trobe University, Melbourne, VIC Australia; Central Clinical School, Monash University, Melbourne, VIC Australia; Melbourne Sexual Health Centre, Alfred Health, Melbourne, VIC Australia; Sydney Sexual Health Centre, Sydney Hospital, Sydney, NSW Australia; Centre for Social Research in Health, UNSW Australia, Sydney, NSW Australia; Victorian AIDS Council/Gay Men’s Health Centre, Melbourne, VIC Australia; ACON, Sydney, NSW Australia; Cairns Sexual Health Service, Cairns North, QLD Australia; James Cook University, Townsville, QLD Australia

**Keywords:** HIV, MSM, Testing, Self-test, Home test

## Abstract

**Background:**

Gay and bisexual men (GBM) are a major risk group for HIV acquisition, yet the majority of higher-risk GBM test for HIV less often than recommended (3–6 monthly). HIV self-testing has the potential to increase testing frequency and improve awareness of personal HIV status. HIV self-tests have been approved in some countries, however there are concerns whether self-testing would increase HIV testing frequency enough to compensate for the reduced sensitivity of self-tests in early infection. We describe here a randomised controlled trial to assess the effectiveness of self-testing in increasing HIV testing frequency among higher-risk GBM, and its acceptability.

**Methods/design:**

Participants are higher-risk HIV negative GBM (>5 partners or condomless anal intercourse in previous 3 months; n = 350), including 50 GBM who tested for HIV over two years ago or never tested before (‘infrequent-testers’). Participants are recruited from sexual health clinics and community-based organisations, and randomised 1:1 to either self-testing or standard-care (routine clinic-based testing) arms. The trial employs a wait-list control design: participants in the standard-care arm switch to self-testing arm in the second year, and gain access to self-test kits. Participants in the self-testing arm receive four oral-fluid self-test kits at enrolment, with additional kits provided on request. Demographics, sexual behaviour and HIV testing preferences are collected at baseline, and the frequency and pattern of HIV and sexually transmissible infection (STI) testing is collected via online 3-monthly questionnaires. The acceptability of self-testing is assessed at 12 months via an online questionnaire and in-depth interviews. A 24-h telephone support is provided, with expedited follow-up of those with reactive self-test results. The primary outcome is HIV testing frequency (mean number of HIV tests per person) over 12 months, and the secondary outcomes are: mean number of STI tests (chlamydia, gonorrhoea, syphilis) per person; reasons for HIV testing; and acceptability of HIV self-testing.

**Discussion:**

This is the first trial to evaluate the use of self-testing among GBM in Australia, and the first internationally among infrequent testers. The study will provide evidence on whether self-testing increases HIV testing frequency, and its acceptability among GBM. The findings will improve our understanding of self-testing patterns, and whether GBM supplement or replace their existing testing routine.

**Trial registration:**

Australian and New Zealand Clinical Trial Registration number: ACTRN12613001236785, registered on November 12, 2013.

## Background

Gay and bisexual men (GBM) are a major risk group for HIV acquisition, and are disproportionately affected by HIV in many countries [1]. The rates of HIV diagnoses among GBM in high income countries including the United States (US), United Kingdom (UK), and Australia have increased over the past decade [2–5]. In Australia, annual HIV diagnoses have increased by about 70 % since 1999, and over two-thirds of new infections are diagnosed in GBM [5].

Regular testing is recognised as a key strategy for HIV control. Reduction in risky sexual practices as a result of the awareness of one’s HIV-positive status [6–8], and early initiation of antiretroviral therapy, can substantially reduce the risk of HIV transmission to sexual partners [9–11]. Mathematical modelling suggests that earlier diagnosis and treatment of HIV-positive individuals could substantially reduce population incidence [12]. A recent trial also confirmed individual benefits with early initiation of antiretroviral therapy [13]. Clinical guidelines in many countries recommend that sexually active GBM should have at least annual HIV testing, with 3–6 monthly testing for higher-risk men [14].

HIV testing uptake among GBM in many middle and high income countries is lower than the recommended level [15–19], and less than a quarter of higher-risk Australian GBM test every 3–6 months consistent with the guidelines [20]. Between 8 and 34 % of HIV-positive GBM in Australia [21, 22], New Zealand [23], US [24, 25], UK [18], and Canada [26] are unaware of their HIV-positive status. Mathematical modelling suggests that individuals with undiagnosed HIV infections contribute disproportionately to HIV transmission [27, 28]. A number of barriers to frequent HIV testing among GBM have been identified, including: perceptions of being at low risk, fear of an HIV-positive diagnosis, no symptoms or illness, and structural barriers such as the need to return to clinics for test results, facing difficulties with appointments, lack of time, and the cost and inconvenience associated with attending clinics [17, 29, 30].

HIV self-testing has the potential to increase testing uptake among GBM by overcoming some of the impediments to testing, particularly structural factors, improve awareness of personal HIV-status, and give a greater sense of control over men’s own health [31, 32]. HIV self-testing kits have been approved for sale in the US [33] and UK [34], and other countries including France and Kenya have also changed regulations to allow self-testing for HIV [33]. Australian HIV testing policy was changed in July 2014 to support self-testing for HIV, allowing manufactureres to submit applications to the Therapeutics Goods Administration for approval of self-tests [35], however no self-tests have yet been approved for sale.

Surveys of GBM in Australia [36–40] and overseas indicate that men are highly interested in accessing self-testing if available [17, 41–46], and likely to increase their HIV testing frequency [47]. However, concerns remain about errors in interpreting test results by users, relatively lower sensitivity in early infections, emotional consequences of reactive results and access to counselling, impact on STI testing frequency, and linking people diagnosed with a self-test to care [32, 48, 49]. HIV self-testing has been found to be highly acceptable in a range of settings and populations [50–52], with little or no evidence of harm associated with self-testing (including anxiety, fear, worry, suicide, or harm as a result of false results) [53]. A high proportion of GBM in observational self-testing studies report that self-testing is easy to perform, and express willingness to test again using self-tests [54–58]. Almost all individuals (96 %) diagnosed with HIV in an unobserved field evaluation of oral fluid self-testing in the US said they would access follow-up confirmatory testing [59].

An oral fluid self-test (the OraQuick In-Home HIV Test) was approved in the US on public health grounds despite its relatively lower sensitivity in the field trials [32], and acute HIV infections [60]. It was estimated that in the first year of its availability, 2.8 million people in the US would use the OraQuick In-Home HIV Test, and even though there would be 3800 false-negative test results (missed infections), this would be offset by 45,000 new infections being detected. Overall a reduction of more than 4000 new HIV transmissions in the first year was estimated [32]. However, a subsequent modelling study predicted that if used by GBM in Seattle, HIV prevalence would increase as men would replace conventional laboratory tests at clinics with a less sensitive self-test [61]. It is worth noting that the model had important limitations, for example, not including men who had not tested before and assuming men would replace their routine testing with self-tests rather than any supplementation [62]. A recent analysis based on the Australian epidemic demonstrated that there would be a public health benefit (detection of HIV infections that would have otherwise remained undiagnosed) from self-testing if higher-risk GBM supplemented clinic-based testing using the OraQuick self-test or previously untested GBM used a self-test [63].

To date, there is evidence from only one, as yet unpublished, randomised controlled trial (RCT) in Seattle that access to HIV self-testing increases HIV testing frequency among GBM [47]. However, to our knowledge, no studies have specifically evaluated the use of HIV self-testing in GBM who test relatively infrequently or have never tested before. We describe here a randomised controlled trial which aims to assess the effectiveness and acceptability of HIV self-testing in higher-risk GBM who test more frequently (last HIV test within the past two years) and less frequently (last HIV test over two years ago or never tested before).

## Methods

### Study design

**Fig. 1:**
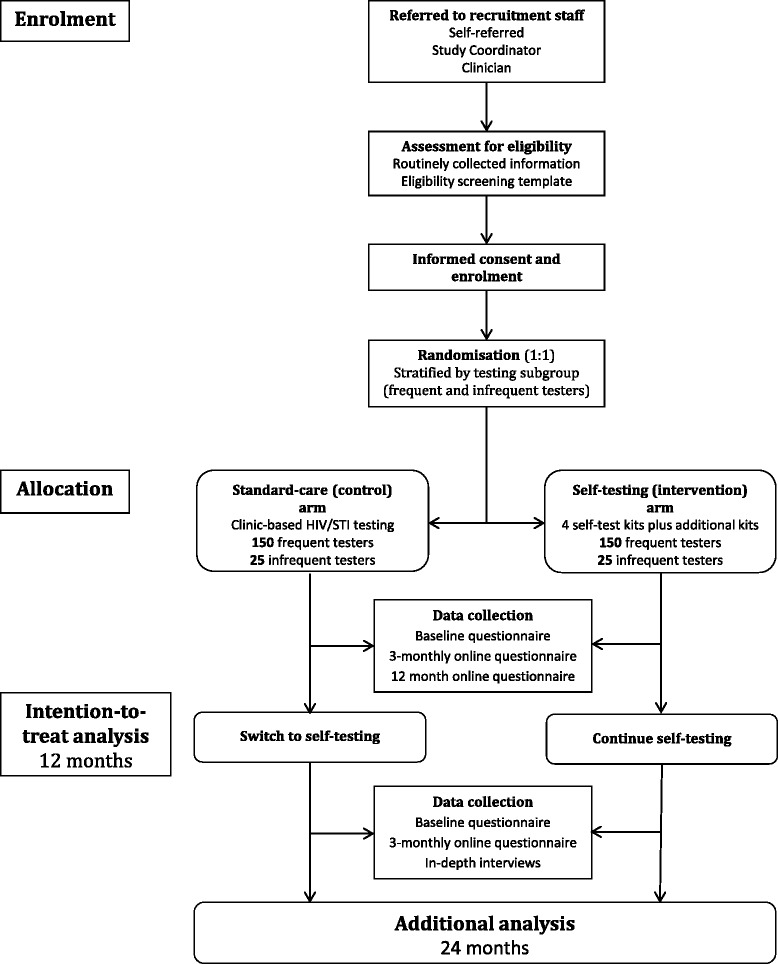
Trial overview

This is a non-blinded wait-list control RCT with individuals randomised in a 1:1 ratio to two study arms: intervention (HIV self-testing) arm; and control (standard-care) arm. Participants in each arm will be followed-up for two years. Participants in the standard-care arm will switch to the self-testing arm after one year of follow-up and get access to self-testing in the second year (Fig. 1).

### Participants

Participants include two testing subgroups: those who had their last HIV test within the past two years (hereafter called ‘frequent testers’) and those who had their last HIV test over two years ago, or have never tested before (hereafter called ‘infrequent testers’).

### Study objectives

The primary objective of this trial is to compare HIV testing frequency between the self-testing and standard-care arms. The secondary objectives are to compare the frequency of sexually transmissible infection (STI) testing including chlamydia, gonorrhoea and syphilis between the two study arms; and to assess the reasons for HIV testing and acceptability of HIV self-testing in the self-testing arm.

### Study duration

The study will require 3 years to complete: 6 months for recruitment, 24 months to complete the follow-up and data collection, and 6 months for analysis and reporting.

### Ethical considerations

The study protocol has been approved by the South Eastern Sydney Local Health District Human Research Ethics Committee (HREC) and the Alfred Hospital HREC. Additional ethical approvals have been obtained from Victorian AIDS Council Research Promotion and Ethics Committee, and ACON Research Ethics Review Committee.

### Inclusion and exclusion criteria

Participants are eligible if they: identify as gay or bisexual men; are aged 18 years or above; are HIV negative (either self-reported or based on clinical records); plan to live in Australia for the next 2 years; and report any condomless anal intercourse, or >5 male sexual partners in the past 3 months. The eligibility criteria are adopted from Australian clinical testing guidelines to recruit higher-risk GBM [64], as they have the highest HIV incidence in Australia [65]. Participants are excluded if they: cannot speak and/or read English; cannot provide written consent; or are unwilling or unable to comply with all the requirements of the study.

### Recruitment

Participants are recruited from large urban sexual health centres and community-based organisations in Sydney, Melbourne and Cairns, including: Sydney Sexual Health Centre, Melbourne Sexual Health Centre, Cairns Sexual Health Service, ACON Sydney (the largest gay men’s health organisation in New South Wales), and the Victorian AIDS Council, Melbourne. Promotion of the study occurs through gay media, the study website [66] and websites of participating organisations, social media (Facebook), advertisements on Grindr (a mobile device social networking application for GBM), and posters and postcards at recruitment sites. Potentially eligible participants are informed about the study by recruitment staff and clinicians. The clinical sites also use other strategies for engaging potential participants, for example: eligible participants receive a prompt when they complete a computer-assisted self-interview for routine data collection; and study nurse receives an SMS (text message) when an eligible patient completes a computer-assisted self-interview. Participants are also able to express interest in the study by completing an online form via the study website. The study coordinator assesses the information provided and refers eligible participants to the recruitment sites.

All participants are required to attend one of the study sites for recruitment. A dedicated study nurse at each clinic or a designated staff member at the community sites confirms participants’ eligibility via routinely collected data on sexual behaviour or by using a paper-based eligibility screening template. Those eligible are given an information sheet, and the study procedures are explained to them. It is emphasised that the self-test is not recommended for use within three months of a potential exposure or risk event (the window period), and that reactive self-test results must be confirmed by a conventional laboratory test at a clinic. Participants are required to sign a written informed consent at recruitment covering all trial procedures and data collection.

### Randomisation

Computer-generated randomisation codes, stratified by testing group (frequent and infrequent testers), are produced by a biostatistician and sealed in opaque envelopes by a research assistant not associated with the trial. Once written consent is obtained, the recruitment staff select the next randomisation envelope according to the participant’s testing group and inform the participant of the study arm they are being assigned to – the self-testing or standard-care arm.

### Blinding

Given the nature of the intervention, it is not feasible to blind the recruitment staff and participants to their study arm allocation. However, recruitment staff are unaware of the allocation until the envelope is opened in front of the participant. The statistician analysing RCT data will be blinded to the study arms.

### Study procedures

#### Intervention (self-testing) arm

Participants randomised to the self-testing arm have access to oral fluid HIV self-test kits in addition to the usual clinic-based HIV/STI testing and care. At enrolment, participants in the self-testing arm receive four self-test kits including the manufacturer-supplied step-by-step instructions and a web-link to an instructional video. Four self-test kits provide a 12 month supply for higher-risk men who test every 3–6 months consistent with the guidelines [64]. Participants are able to request additional kits, one at a time, for a maximum of 12 kits in one year in case repeat testing is needed or men test more frequently in relation to risky sexual behaviour. Depending on participants’ preference, additional self-test kits are either picked-up from the study sites or sent by post in plain packaging. At the beginning of the second year of follow-up, all participants (including those switching from standard-care to the self-testing arm) are eligible to collect four self-test kits from study sites, or sent by post if requested. A central log of kits dispatched is maintained to keep track of the number of kits supplied to each participant. No training on performing self-tests is provided at baseline to mimic a real-world scenario where untrained individuals will purchase self-test kits over-the-counter from pharmacies or online.

##### HIV self-testing kits

**Fig. 2:**
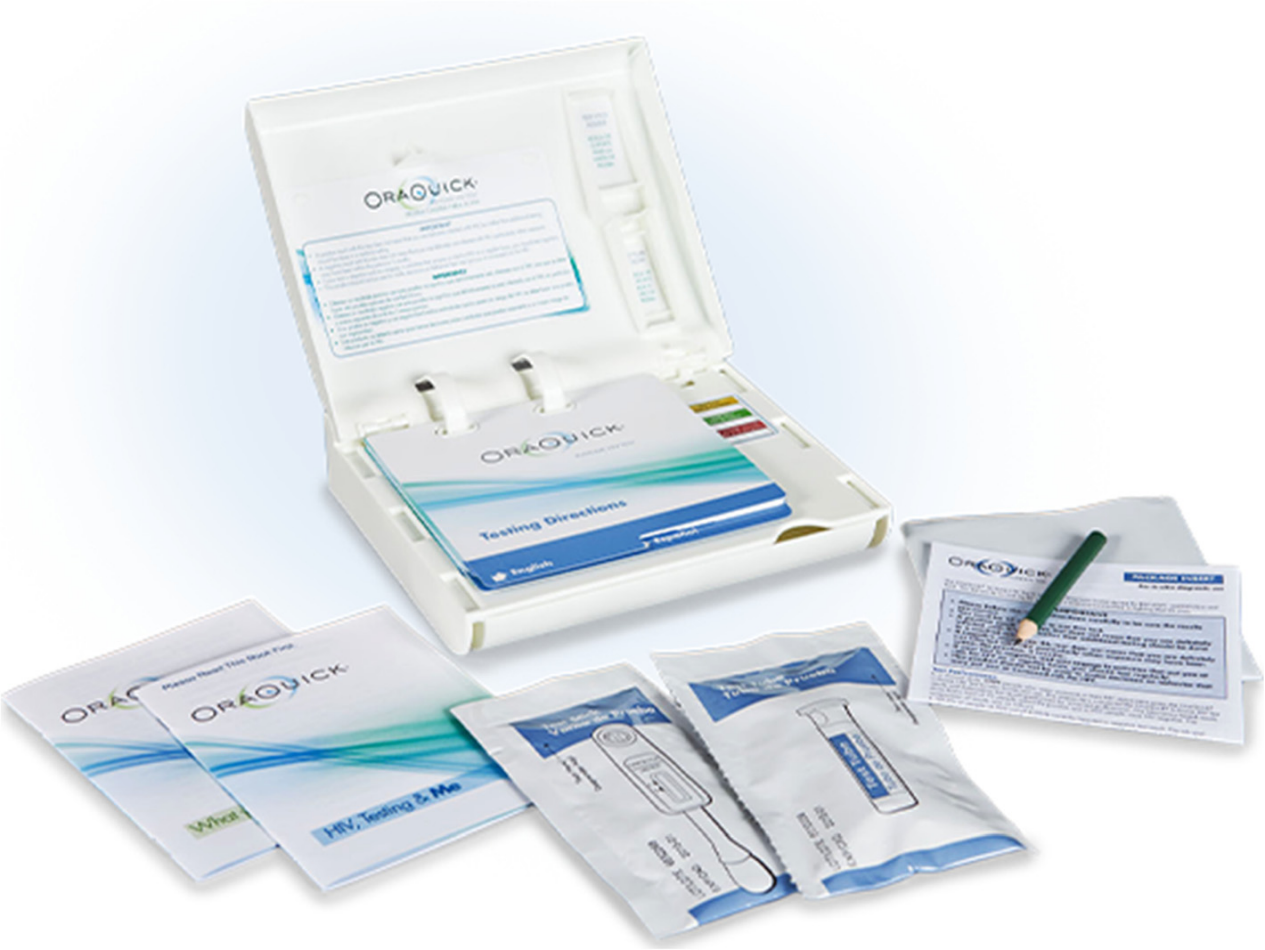
OraQuick In-Home HIV Test kit contents: instruction booklet (flipchart design), specimen collection device, developer solution vial, pre-test and post-test information booklets, pencil, and disposal bag (Source: http://www.oraquick.com)

The US Food and Drug Administration approved ‘OraQuick In-Home HIV test’ (OraSure Technologies Inc., Bethlehem, PA, USA) is being used for HIV self-testing in this study (Fig. 2). It is a second generation test which detects HIV 1/2 antibodies in oral fluid specimens. The specimen is obtained by swabbing the upper and lower gums using a collector device which is then transferred to a solution, and the results are read in 20–40 min [59]. The sensitivity and specificity of the OraQuick self-test in the hands of untrained users is 91.7 % (95 % confidence interval [CI]: 84.2-96.3 %) and 99.9 % (95 % CI: 99.9-100.0 %), respectively [59]. Every OraQuick kit is accompanied by step-by-step graphic and written instructions and two booklets with general information on HIV testing and interpretation of results. The manufacturer-specified window period for OraQuick self-test is three months [59], however published data show that second generation antibody immunoassays detect HIV antibodies within 25–35 days after HIV infection [67, 68]. The OraQuick self-test is a preliminary test and all reactive results must be confirmed with a conventional laboratory test. As the test kit is packaged for the US market, each self-test kit supplied to participants is prominently labelled with Australian telephone numbers for support and emergencies.

##### Telephone support lines

A 24-h telephone support line staffed by the study coordinators on a rotation basis is available to participants, providing support for the use of self-test kits, interpretation of results, and advice for reactive home test results. In addition, participants have access to existing telephone support lines of participating sexual health centres. A log of calls to the telephone support lines is maintained and the reason for each call is recorded.

##### Follow-up of reactive self-tests

Participants are advised to inform the study coordinator of any reactive self-test results. The responses to 3-monthly online questionnaires (see below) are also monitored for any reported reactive self-test results. Any participant with a reactive self-test result is advised by the study coordinator to undergo confirmatory laboratory testing and offered expedited clinical review and supportive counselling at the study clinics. If they prefer to go to a clinic other than the study clinics, they are offered assistance in arranging an appointment. The laboratory confirmation and management of participants with confirmed HIV diagnoses will be based on the standard protocols at the respective clinic, however their study involvement will cease.

#### Control (standard-care) arm

Participants randomised to the standard-care arm continue to access routine clinic-based HIV/STI testing and care at their preferred clinic, including clinics other than the study clinics. In Australia, most laboratories use fourth generation HIV screening immunoassays [69]. Supplementary HIV antibody, HIV p24 antigen, and Western Blot testing are performed on specimens that are reactive by the screening assay, and deemed positive if consistent with the national case definition [70]. After 12 months, participants in the standard-care arm switch to the self-testing arm and follow the same procedures as the self-testing arm.

### Outcome measures

The primary outcome is the mean number of HIV tests per person over 12 months. The secondary outcomes are: the mean number of STI (chlamydia, gonorrhoea, syphilis) tests per person; reasons for HIV testing; and the acceptability of HIV self-testing.

### Data collection

#### Clinical data

HIV and STI testing data for the duration of the study will be extracted from the patient management system for all participants recruited at study clinics.

#### Surveys

##### Baseline

All participants complete a self-administered online survey on a handheld electronic device at enrolment. The survey covers socio-demographics, sexual risk behaviour, HIV/STI testing history, attitudes to HIV self-testing, opinions about treatments for HIV, and an HIV testing self-efficacy scale measuring gay men's confidence in their ability to undertake HIV testing [71].

##### 3-monthly

Participants in both arms of the study complete a brief online survey every three months asking the number, location, reasons for, and results of HIV self-tests and HIV/STI tests at clinics and community sites, and sexual risk behaviour since their last survey. Participants are also asked about the number of self-test kits they used to test their sexual partner(s) or gave to someone else to test at another time. If participants report episodes of HIV/STI testing at clinics other than study clinics, those clinics are contacted to obtain test results. Consent for release of results is obtained at enrolment and mailed/faxed to relevant clinics when obtaining results.

##### 12 months

At 12 months, a survey is conducted including the same questions as the 3-monthly survey with some additional questions about self-testing acceptability, ease of use overall and for various steps of HIV self-testing, participants’ experience using self-tests to test themselves and their partner(s), if relevant, and their preferences for accessing and using HIV self-testing in the future.

#### Survey reminders

If participants have not completed a survey, they are sent up to three reminders via email or SMS, each one week apart. If surveys are not completed after three reminders, participants are sent a final SMS asking the total number of HIV tests and the number of HIV self-tests since their last survey.

#### In-depth interviews

About 25 participants in the self-testing arm will be invited to participate in a face-to-face semi-structured in-depth interview at the completion of 12 months follow-up. Participants are asked at enrolment if they are willing to be contacted for in-depth interviews. The interviews will focus on collecting detailed information about their HIV testing pattern prior to the study, their experience using self-tests to test themselves and their partner(s) during the study, any changes in their testing patterns and sexual behaviour after getting access to self-testing, and their opinions about HIV self-testing in the future. Participants from each of the following groups will be interviewed: those who tested themselves using self-tests only; those who tested at clinics and supplemented their testing with self-tests; those who tested their partner(s) using self-tests; and those who had a confirmed positive or false-reactive self-test result.

### Statistical analysis plan

All analyses will be conducted using Stata 14. The mean HIV and STI testing frequency in each arm (self-testing and standard-care) by testing subgroup (frequent and infrequent testers) will be assessed using an intention-to-treat analysis at 12 months. The per-protocol analyses will include calculating: (i) the reasons for HIV testing; and (ii) the acceptability of HIV self-testing in the self-testing arm.

The mean HIV testing frequency per person in the first 12 months will be calculated, excluding any tests performed at enrolment. Only self-reported HIV tests where there is a record of an HIV test occurring at a clinic or community site will be included. The following tests will be excluded from the testing frequency calculation: HIV self-tests where participants did not obtain a result for any reason; self-test kits used to test a partner(s) or given to someone else; and confirmatory HIV tests following a reactive self-test. STI testing will include episodes of testing for chlamydia, gonorrhoea, or syphilis tests, and will be included where there is a record of a test occurring at a clinic or community site.

The effects of the intervention will be measured by comparing the mean HIV testing frequency, the mean STI testing frequency, and mean clinic-based HIV tests in each arm by testing subgroup using a *t*-test. The reasons for self-tests and clinic tests in the self-testing arm will be compared using repeated measures logistic regression clustered on individual. The acceptability analysis will be descriptive only. Differences in proportions will be investigated using Chi^2^ statistics.

### Sample size

In the analysis, we intend to assess the primary outcome for the frequent and infrequent testers subgroups and overall. For frequent testers, a sample size of 112 in each arm will detect an increase in HIV testing frequency from 1.5 tests per year to 2 tests per year (standard deviation [SD]: 2.4) with 80 % statistical power and type I error probability of 0.05. The frequency of 1.5 tests per year is based on the results of a previous clinic based RCT in Melbourne [72], and data from Australian sexual health clinics [73]. For infrequent testers, a sample size of 14 in each arm will detect an increase in HIV testing frequency from 0.2 tests per year (assuming one HIV test in the past five years based on clinic data [73]) to 1 test per year (SD: 0.7) with 80 % statistical power and type I error probability of 0.05. On this basis, we conservatively estimate a sample size of 300 frequent testers and 50 infrequent testers for an overall sample size of 350. The sample size of 350 will achieve at least 80 % statistical power to detect an overall increase from 1.5 to 2 tests per year (SD: 2.4) for the two groups combined.

### Trial registration

The trial is registered with the Australian and New Zealand Clinical Trial Registry [trial ID: ACTRN12613001236785, registered on November 12, 2013].

## Discussion

Gay and bisexual men are at an increased risk of acquiring HIV infection, and the rates of HIV diagnoses in GBM have increased in recent years. Yet, the uptake of HIV testing among GBM in Australia and other countries is lower than the recommended level, and a significant proportion of HIV infections remain undiagnosed. HIV self-testing has the potential to improve testing uptake and frequency among GBM. To our knowledge, the FORTH trial is the first in Australia to assess whether access to HIV self-testing increases HIV testing frequency among GBM, and the first internationally among GBM who test less frequently or have never tested before.

A major strength of the FORTH trial is the focus on infrequent testers. A recent Australian study showed that in addition to GBM using self-tests to supplement their existing testing, there will be a public health benefit if previously untested GBM test for the first time using HIV self-tests [63]. In Australia, about 20 % of GBM in community-based samples reported their last HIV test was over two years ago or have never tested for HIV [19]. In 2011, about 17 % of GBM in six US cities reported they had never tested before for HIV [17]. An RCT in Seattle showed that access to HIV self-testing significantly increased HIV testing frequency among GBM over a 15 months period (3.6 vs. 5.3 tests per person) [47], however the study did not specifically explore the outcomes in infrequent testers. In addition, there are other methodological differences: our sample size is larger (350 vs. 230); follow-up duration is longer (24 months vs. 15 months) allowing us to assess the sustainability of the intervention; and the wait-list design will allow us to assess the impact of delayed access to self-testing on HIV testing frequency.

An important consideration when GBM start using self-tests is whether they supplement or replace their routine testing with self-tests [37]. Though cross-sectional surveys of Australian GBM indicate that they are likely to test more often if self-testing is available [36], our study will provide conclusive evidence as to whether access to HIV self-testing among GBM: increases testing frequency, and to a level where a public health benefit can be achieved [63]; leads men to supplement or replace their existing testing; and leads untested men to test for HIV for the first time. Also, the findings will improve our understanding of the reasons for self-testing among GBM, and whether men use self-tests to test themselves after risk events. We are also asking participants about their preferences for future self-testing delivery methods including online, pharmacy, and clinics, to understand how men would prefer to access self-testing when available.

A major concern in relation to HIV self-testing is that it may lead to GBM testing less frequently for other STIs including chlamydia, gonorrhoea and syphilis, for which they would still need to attend clinics. The prevalence of STIs is high in GBM, and repeat rectal chlamydia and gonorrhoea infections have been associated with an increased risk of HIV seroconversion [74]. Our study will be able to assess whether access to HIV self-testing will lead to a lower STI testing frequency in GBM.

Previous studies have indicated that GBM are willing to use self-tests to test partners before sexual intercourse, and there is potential for detecting undiagnosed infections [75]. Although not a main outcome of this study, we are collecting information about the number of self-test kits participants used to test their partners and the type of partners they tested. This will be further explored in in-depth interviews to contextualise the use of self-tests with partners, for example, whether men use self-tests to negotiate safe sex, make decisions to have sex without condoms and so on.

The study will contribute to the scarce literature internationally on the impact of self-testing on HIV testing frequency among GBM. The study will provide evidence on the level of testing that could be achieved with HIV self-testing among Australian GBM, and whether men supplement or replace their existing testing with self-testing. The findings will improve our understanding of the safety, effectiveness and acceptability of self-testing, particularly among infrequent testers. The findings will have important implications for self-testing policy, both in Australia and internationally.

## Abbreviations

FORTH: Frequency of Oral Rapid Testing at Home
GBM: Gay and bisexual men
HREC: Human Research Ethics Committee
NHMRC: National Health & Medical Research Council
RCT: Randomised controlled trial
SD: Standard deviation
STI: Sexually transmissible infections
UK: United Kingdom
US: United States

## References

[1] Beyrer C, Baral SD, van Griensven F, Goodreau SM, Chariyalertsak S, Wirtz AL (2012). Global epidemiology of HIV infection in men who have sex with men. Lancet.

[2] Centres for Disease Control and Prevention. HIV surveillance report, 2010, vol 22. 2012. http://www.cdc.gov/hiv/pdf/statistics_surveillance_report_vol_22.pdf. Accessed 2 Oct 2015.

[3] Health Protection Agency (2011). HIV in the United Kingdom: 2011 Report.

[4] Sullivan PS, Hamouda O, Delpech V, Geduld JE, Prejean J, Semaille C (2009). Reemergence of the HIV epidemic among men who have sex with men in North America, Western Europe, and Australia, 1996–2005. Ann Epidemiol.

[5] The Kirby Institute (2014). HIV, viral hepatitis and sexually transmissible infections in Australia annual surveillance report 2014: The Kirby Institute.

[6] Fox J, White PJ, Macdonald N, Weber J, McClure M, Fidler S (2009). Reductions in HIV transmission risk behaviour following diagnosis of primary HIV infection: a cohort of high-risk men who have sex with men. HIV Med.

[7] Gorbach PM, Weiss RE, Jeffries R, Javanbakht M, Drumright LN, Daar ES (2011). Behaviors of recently HIV-infected men who have sex with men in the year post-diagnosis: effects of drug use and partner types. J Acquir Immune Defic Syndr.

[8] Marks G, Crepaz N, Senterfitt JW, Janssen RS (2005). Meta-analysis of high-risk sexual behavior in persons aware and unaware they are infected with HIV in the United States: implications for HIV prevention programs. J Acquir Immune Defic Syndr.

[9] Grulich AE, Bavinton B, Jin FY, Prestage G, Zablotska I, Koelsch K (2015). HIV transmission in male serodiscordant couples in Australia, Thailand and Brazil.

[10] Cohen MS, Chen YQ, McCauley M, Gamble T, Hosseinipour MC, Kumarasamy N (2011). Prevention of HIV-1 infection with early antiretroviral therapy. N Engl J Med.

[11] Rodger A, Bruun T, Cambiano V, Vernazza P, Estrada V, Van Lunzen J (2014). HIV transmission risk through condomless sex if HIV+ partner on suppressive ART: PARTNER study.

[12] Jansson J, Kerr CC, Wilson DP (2013). Predicting the population impact of increased HIV testing and treatment in Australia. Sex Health.

[13] The INSIGHT START Study Group. Initiation of antiretroviral therapy in early asymptomatic HIV infection. N Engl J Med. 2015; doi:10.1056/NEJMoa1506816. 10.1056/NEJMoa1506816 PMC4569751 26192873

[14] Patel RR, Patel S, Clarke E, Khan AW, Doshi B, Radcliffe KW (2014). Guidance and practice on frequency of HIV and sexually transmitted infection testing in men who have sex with men – what is the European situation?. Int J STD AIDS.

[15] Chow EPF, Wilson DP, Zhang L (2012). The rate of HIV testing is increasing among men who have sex with men in China. HIV Med.

[16] Kerr LRFS, Mota RS, Kendall C, Pinho AA, Mello MB, Guimarães MDC (2013). HIV among MSM in a large middle-income country. AIDS.

[17] MacKellar DA, Hou S-I, Whalen CC, Samuelsen K, Sanchez T, Smith A (2011). Reasons for not hiv testing, testing intentions, and potential use of an over-the-counter rapid HIV test in an internet sample of men who have sex with men who have never tested for hiv. Sex Transm Dis.

[18] Wayal S, Parsons V, Copas A, Hart G, Gilson R, Johnson A (2015). Trends in undiagnosed HIV and HIV testing behaviour in community samples of men who have sex with men in London, UK: Results from repeat cross-sectional surveys between 2000–2013. HIV Med.

[19] Zablotska I, Holt M, De Wit J, McKechnie M, Mao L, Prestage G (2012). Gay men who are not getting tested for HIV. AIDS Behav.

[20] Guy R, Goller JL, Spelman T, El-Hayek C, Gold J, Lim M (2010). Does the frequency of HIV and STI testing among men who have sex with men in primary care adhere with Australian guidelines?. Sex Transm Infect.

[21] Holt M, Lea T, Asselin J, Hellard M, Prestage G, Wilson D (2014). The prevalence of undiagnosed HIV infection is lower than expected among gay and bisexual men in four Australian cities: preliminary findings from the COUNT study.

[22] Mallitt K-A, Wilson DP, McDonald A, Wand H (2012). HIV incidence trends vary between jurisdictions in Australia: an extended back-projection analysis of men who have sex with men. Sex Health.

[23] Saxton PJ, Dickson NP, Griffiths R, Hughes AJ, Rowden J (2012). Actual and undiagnosed HIV prevalence in a community sample of men who have sex with men in Auckland. New Zealand BMC Public Health.

[24] Raymond HF, Chen Y-H, Ick T, Scheer S, Bernstein K, Liska S (2013). A new trend in the HIV epidemic among men who have sex with men, San Francisco, 2004–2011. J Acquir Immune Defic Syndr.

[25] Wejnert C, Le B, Rose CE, Oster AM, Smith AJ, Zhu J (2013). HIV infection and awareness among men who have sex with men–20 cities, United States, 2008 and 2011. PLoS One.

[26] Moore DM, Kanters S, Michelow W, Gustafson R, Hogg RS, Kwag M (2011). Implications for HIV prevention programs from a serobehavioural survey of men who have sex with men in Vancouver, British Columbia: the ManCount study. Can J Public Health.

[27] Marks G, Crepaz N, Janssen RS (2006). Estimating sexual transmission of HIV from persons aware and unaware that they are infected with the virus in the USA. AIDS.

[28] Wilson DP, Hoare A, Regan DG, Law MG (2009). Importance of promoting HIV testing for preventing secondary transmissions: modelling the Australian HIV epidemic among men who have sex with men. Sex Health.

[29] Conway D, Holt M, Couldwell D, Smith D, Davies D, McNulty A (2015). Barriers to HIV testing and characteristics associated with never testing among gay and bisexual men attending sexual health clinics in Sydney. J Int AIDS Soc.

[30] Prestage G, Brown G, Keen P (2012). Barriers to HIV testing among Australian gay men. Sex Health.

[31] Campbell S, Klein R (2006). Home testing to detect human immunodeficiency virus: boon or bane?. J Clin Microbiol.

[32] Myers JE, El-Sadr WM, Zerbe A, Branson BM (2013). Rapid HIV self-testing: long in coming but opportunities beckon. AIDS.

[33] Wong V, Johnson C, Cowan E, Rosenthal M, Peeling R, Miralles M (2014). HIV self-testing in resource-limited settings: regulatory and policy considerations. AIDS Behav.

[34] The Lancet HIV (2015). Making the most of a new HIV self-test. Lancet HIV.

[35] Commonwealth of Australia (2014). Seventh national HIV strategy.

[36] Bavinton B, Brown G, Hurley M, Bradley J, Keen P, Conway D (2013). Which gay men would increase their frequency of HIV testing with home self-testing?. AIDS Behav.

[37] Bilardi JE, Walker S, Read T, Prestage G, Chen MY, Guy R (2013). Gay and bisexual men’s views on rapid self-testing for HIV. AIDS Behav.

[38] Chen MY, Bilardi JE, Lee D, Cummings R, Bush M, Fairley CK (2010). Australian men who have sex with men prefer rapid oral HIV testing over conventional blood testing for HIV. Int J STD AIDS.

[39] Hull P, Holt M, Mao L, Kao S, Prestege G, Zablotska I (2011). Gay community periodic survey: Sydney February 2011.

[40] Lee E, Holt M, Mao L, McKanzie T, Batrouney C, Kennedy M (2011). Gay community periodic survey: Melbourne 2011.

[41] Greacen T, Friboulet D, Blachier A, Fugon L, Hefez S, Lorente N (2013). Internet-using men who have sex with men would be interested in accessing authorised HIV self-tests available for purchase online. AIDS Care.

[42] Rosales-Statkus ME, de la Fuente L, Fernández-Balbuena S, Figueroa C, Fernàndez-López L, Hoyos J (2015). Approval and potential use of over-the-counter HIV self-tests: the opinion of participants in a street based HIV rapid testing program in Spain. AIDS Behav.

[43] Lippman SA, Périssé AR, Veloso VG, Sullivan PS, Buchbinder S, Sineath RC (2014). Acceptability of self-conducted home-based HIV testing among men who have sex with men in Brazil: data from an on-line survey. Cad Saude Publica.

[44] Saunders JM, Mercer CH, Sutcliffe LJ, Hart GJ, Cassell J, Estcourt CS. Where do young men want to access STI screening? A stratified random probability sample survey of young men in Great Britain. Sex Transm Infect. 2012; doi:10.1136/sextrans-2011-050406. 10.1136/sextrans-2011-050406 PMC3461759 22510331

[45] Sharma A, Sullivan PS, Khosropour CM (2011). Willingness to take a free home HIV test and associated factors among internet-using men who have sex with men. J Int Assoc Provid AIDS Care.

[46] Wong H, Tam H, Chan D, Lee S (2015). Usage and acceptability of HIV self-testing in men who have sex with men in Hong Kong. AIDS Behav.

[47] Katz D, Golden M, Hughes J, Farquhar C, Stekler J (2015). HIV self-testing increases HIV testing frequency among high-risk men who have sex with men: a randomized controlled trial.

[48] Koval CE (2012). Home testing for HIV: Hopefully, a step forward. Cleve Clin J Med.

[49] Walensky RP, Bassett IV (2011). HIV self-testing and the missing linkage. PLoS Med.

[50] Krause J, Subklew-Sehume F, Kenyon C, Colebunders R (2013). Acceptability of HIV self-testing: a systematic literature review. BMC Public Health.

[51] Pai NP, Sharma J, Shivkumar S, Pillay S, Vadnais C, Joseph L (2013). Supervised and unsupervised self-testing for HIV in high-and low-risk populations: A systematic review. PLoS Med.

[52] Figueroa C, Johnson C, Verster A, Baggaley R. Attitudes and acceptability on HIV self-testing among key populations: A literature review. AIDS Behav. 2015;1–17. 10.1007/s10461-015-1097-8 PMC4598350 26054390

[53] Brown A, Djimeu E, Cameron D (2014). A review of the evidence of harm from self-tests. AIDS Behav.

[54] Choko AT, Desmond N, Webb EL, Chavula K, Napierala-Mavedzenge S, Gaydos CA (2011). The uptake and accuracy of oral kits for hiv self-testing in high hiv prevalence setting: A cross-sectional feasibility study in Blantyre Malawi. PLoS Med.

[55] de la Fuente L, Rosales-Statkus ME, Hoyos J, Pulido J, Santos S, Bravo MJ (2012). Are participants in a street-based HIV testing program able to perform their own rapid test and interpret the results?. PLoS One.

[56] Gaydos CA, Hsieh Y-H, Harvey L, Burah A, Won H, Jett-Goheen M (2011). Will patients “opt in” to perform their own rapid HIV test in the emergency department?. Ann Emerg Med.

[57] Pant Pai N, Bhargava M, Sharma J, Balram B, Bois C, Joseph L (2012). Will HIV self testing be accepted by low to medium risk educated populations? A pilot cross sectional study in students of McGill University, Montréal. Montréal, Quebec, Canada.

[58] Peck R, Lim J, van Rooyen H, Mukoma W, Chepuka L, Bansil P (2014). What should the ideal HIV self-test look like? A usability study of test prototypes in unsupervised HIV self-testing in Kenya, Malawi, and South Africa. AIDS Behav.

[59] OraSure Technologies (2012). Final advisory committee briefing materials: available for public release.

[60] Stekler JD, O’Neal JD, Lane A, Swanson F, Maenza J, Stevens CE (2013). Relative accuracy of serum, whole blood, and oral fluid HIV tests among Seattle men who have sex with men. J Clin Virol.

[61] Katz DA, Cassels SL, Stekler JD (2014). Replacing clinic-based tests with home-use tests may increase HIV prevalence among Seattle men who have sex with men: evidence from a mathematical model. Sex Transm Dis.

[62] Conway DP, Keen P, Cunningham P, Wilson D (2014). Response to the modeling analysis by Katz et al. on the impact of replacing clinic-based HIV tests with home testing among men who have sex with men in Seattle. Sex Transm Dis.

[63] University of New South Wales and St Vincent’s Centre for Applied Medical Research (UNSW-AMR) HIV Home Testing Assessment Group. Potential public health benefits of HIV testing occurring at home in Australia. Med J Aust. 2015;202(10). 10.5694/mja14.01210 26021364

[64] Templeton DJ, Read P, Varma R, Bourne C (2014). Australian sexually transmissible infection and HIV testing guidelines for asymptomatic men who have sex with men 2014: a review of the evidence. Sex Health.

[65] Guy RJ, Spelman T, Stoove M, El-Hayek C, Goller J, Fairley CK (2011). Risk factors for HIV seroconversion in men who have sex with men in Victoria, Australia: results from a sentinel surveillance system. Sex Health.

[66] FORTH study website. www.forth.org.au.

[67] Branson BM, Stekler JD (2012). Detection of Acute HIV Infection: We can’t close the window. J Infect Dis.

[68] Masciotra S, McDougal JS, Feldman J, Sprinkle P, Wesolowski L, Owen SM (2011). Evaluation of an alternative HIV diagnostic algorithm using specimens from seroconversion panels and persons with established HIV infections. J Clin Virol.

[69] Cunningham P, Downie J, O'Loughlin P, Evans S, Black J (2007). Incremental detection of HIV infections by the HIV antigen/antibody combination assays: An Australian experience. J Med Virol.

[70] Communicable Diseases Network Australia (2013). Australian national notifiable diseases case definition: Human Immunodeficiency Virus.

[71] Jamil MS, Bavinton B, Guy R, Fairley C, Grulich A, Holt M (2015). Confidence in HIV testing ability is associated with higher HIV testing frequency and likelihood to self-test among gay and bisexual men.

[72] Read TRH, Hocking JS, Bradshaw CS, Morrow A, Grulich AE, Fairley CK (2013). Provision of rapid HIV tests within a health service and frequency of HIV testing among men who have sex with men: randomised controlled trial. BMJ.

[73] Guy RJ, Kong F, Goller J, Franklin N, Bergeri I, Dimech W (2010). A new national chlamydia sentinel surveillance system in Australia: evaluation of the first stage of implementation. Commun Dis Intell.

[74] Bernstein KT, Marcus JL, Nieri G, Philip SS, Klausner JD (2010). Rectal gonorrhea and chlamydia reinfection is associated with increased risk of hiv seroconversion. J Acquir Immune Defic Syndr.

[75] Carballo-Diéguez A, Frasca T, Balan I, Ibitoye M, Dolezal C (2012). Use of a rapid hiv home test prevents HIV exposure in a high risk sample of men who have sex with men. AIDS Behav.

